# Alcohol consumers with liver pathology rarely display α-synuclein pathology

**DOI:** 10.1007/s00401-024-02772-4

**Published:** 2024-08-01

**Authors:** Sylwia Libard, Fredrik Tamsen, Irina Alafuzoff

**Affiliations:** 1 Department of Pathology, Uppsala University Hospital, 75185 Uppsala, Sweden; 2 Department of Immunology, Genetics and Pathology, Uppsala University, Uppsala, Sweden; 3 Department of Surgical Sciences, Forensic Medicine, Uppsala University, Uppsala, Sweden

**Keywords:** Hyperphosphorylated τ, Amyloid β-protein, α-synuclein, Transactive DNA-binding protein 43, Heavy alcohol consumption, Liver pathology

## Abstract

It has been suggested that alcohol consumption protects against Parkinson's disease (PD). Here we assessed postmortem tissue samples from the brains and livers of 100 subjects with ages at death ranging from 51 to 93. Twenty percent of these subjects were demented. We used standardized assessment strategies to assess both the brain and liver pathologies (LP). Our cohort included subjects with none, mild, moderate, and severe LP caused by alcohol consumption. We noted a significant negative correlation of categorical data between liver steatosis and α-synuclein (αS) in the brain and a significant negative correlation between the extent of liver steatosis and fibrosis and the extent of αS in the brain. There was a significant negative association between the observation of Alzheimer’s type II astrocytes and αS pathology in the brain. No association was noted between LP and hyperphosphorylated τ (HPτ). No significant correlation could be seen between the extent of LP and the extent of HPτ, amyloid β protein (Aβ) or transactive DNA binding protein 43 (TDP43) in the brain. There were significant correlations observed between the extent of HPτ, Aβ, αS, and TDP43 in the brain and between liver steatosis, inflammation, and fibrosis. Subjects with severe LP displayed a higher frequency of Alzheimer’s type II astrocytes compared to those with no, or mild, LP. The assessed protein alterations were not more prevalent or severe in subjects with Alzheimer’s type II astrocytes in the brain. In all cases, dementia was attributed to a combination of altered proteins, i.e., mixed dementia and dementia was observed in 30% of those with mild LP when compared with 13% of those with severe LP. In summary, our results are in line with the outcome obtained by the two recent meta-analyses suggesting that subjects with a history of alcohol consumption seldom develop an α-synucleinopathy.

## Introduction

Two recent large meta-analyses, one including 52 (63 707 subjects with Parkinson’s disease (PD) and 9 817 controls), and the other including 26 (8798 subjects with PD and 15 699 controls) eligible case–control studies report an inverse association between alcohol consumption and Parkinson’s disease (PD) [22, 37]. A review conducted already in 2013 suggested that it is unclear whether alcohol protects against or aggravates aging-related protein alterations seen in the brain [46]. It was recently reported that mixed pathologies, i.e. hyperphosphorylated τ (HPτ), amyloid β-protein (Aβ), α-synuclein (αS), and transactive DNA binding protein 43 (TDP43) are a common alteration in the aging brain [3]. A recent Medline search yielded approximately 2500 publications with the search terms alcohol and amyloid; close to 2,000 with the search terms alcohol and tau, around 300 with the search terms alcohol and synuclein, and 20 with the search terms alcohol and TDP. Thus, there are a substantial number of publications discussing, suggesting, or negating the existence of an association between neurodegenerative proteinopathies and alcohol consumption. Although it is known that heavy alcohol consumption (HAC) leads to severe brain alterations involving all cell types, whether its influence is detrimental or protective regarding HPτ, Aβ, αS, and TDP43 has not been validated [16, 35]. Alcohol is known to influence other organs as well, i.e., digestive tracts and liver. The liver exhibits pathological alterations in most individuals who have been exposed to alcohol in high enough concentrations and for a long enough time [29]. Alterations to be seen in the gastrointestinal tract are various including profound ultrastructural changes seen by both transmission and scanning electron microscopy [12, 45].

The protein, αS, is considered being causative in Lewy Body Diseases (LBD), i.e., PD, PD with dementia (PDD), Dementia with Lewy Bodies (DLB), and Multi System Atrophy (MSA)[27]. Already in 2007, Braak and colleagues demonstrated, through examination of a few postmortem cases, that αS was not only present in the brain but also in ganglion cells in the gut [10]. Results in line with the above were reported in 2022 when gut samples obtained prior to death were assessed in patients with αS in the brain. Interestingly, αS was detected in the gut years before it was seen in the brain [25]. Further in 2021, it was reported that the accumulation of αS was seen within the liver in subjects with αS in the brain, suggesting that the liver has a potential role in the clearance of αS [47].

There are those who support the idea of transneuronal propagation, i.e., seeding of altered proteins, for example, αS, from the gut to the brain via the vagal nerve. However, there are others who remain skeptical regarding the validity of this hypothesis [14, 26, 31, 43]. Moreover, the idea of “seeding” altered proteins has been proposed through other routes. For instance, injecting a seed of Aβ into the peritoneum of mice has been reported to lead to cerebral amyloid angiopathy (CAA) and aggregation of Aβ in the brain [52]. A similar phenomenon has also been suggested for HPτ [48]. Furthermore, it has been suggested that altered proteins, such as Aβ and HPτ, which are significant for aging-related neurodegeneration, accumulate in the pancreas, particularly in subjects with type II diabetes mellitus [36]. However, contradictory reports have also been published, i.e., islet amyloid peptide was observed in the pancreas in diabetics, whereas the above listed HPτ and Aβ were not seen when brain and pancreas tissues were assessed in as many as 148 subjects [30]. Even the fourth aging-related protein alteration, i.e., TDP43, has been implicated as being altered in the periphery. Loss of nuclear TDP43 has been seen in the pancreas of diabetics with Frontotemporal lobar degeneration (FTLD) [6].

Among all the aging-related protein alterations discussed above, HPτ is the most common, followed by Aβ [3]. Excessive levels of these two proteins together are considered causative factors for Alzheimer’s Disease (AD), and the pathology is referred to as AD Neuropathologic Change (ADNC) [11, 20, 38]. Following these two alterations in occurrence is TDP43, which was primarily seen in the brains of subjects with FTLD [34, 41]. In 2014, it was reported that TDP43 is commonly observed in association with ADNC and often found in the aged subjects [3, 4, 23, 24]. Subsequently, in 2019, a new neuropathological entity, i.e., Limbic-predominant Age-related TDP43 encephalopathy (LATE), was introduced [39, 40].

All four altered proteins, HPτ, Aβ, TDP43, and αS, can be visualized in a standardized manner by applying immunohistochemistry (IHC). The severity of these protein alterations is assessed following defined consensus criteria based on the neuroanatomical distribution of pathology [8, 9, 23, 24, 51].

There are significant obstacles in determining how to reliably assess HAC and estimate the extent of exposure to a toxic substance, such as alcohol. Beverages differ, as well as the duration and extent of consumption, and the data obtained via interviews are not reliable. Thus, to securely assess the influence of alcohol on brain tissue in subjects with HAC is, if not impossible, rather difficult. The liver of subjects with HAC exhibits pathological alterations such as steatosis, inflammation, and fibrosis that could be used as a proxy for long-term alcohol intake. In line with the assessment of brain pathologies, as mentioned above, the assessment of alterations in the liver tissue is standardized, with consensus criteria available for estimating the severity of liver pathology (LP) [7, 13, 18].

The objective of this study was to determine whether there is an association between the incidence and/or severity of all protein alterations seen in the aging brain and the severity of the LP.

## Material and methods

All subjects included in the study cohort had been referred for an autopsy to the Uppsala University Hospital, Department of Pathology, to determine the cause of death. For this study, subjects with available archived brain tissue and liver samples were selected. The selection of cases for this study included subjects with a statement in the referral with indications of HAC to ensure various extents of LP within the cohort. Furthermore, subjects with clinical symptoms in line with PD, and PDD/DLB disease were selected to ensure that cases with αS in the brain would be included in the cohort. In total, 100 subjects fulfilled the inclusion criteria (Table 1), i.e., brain and liver tissue were available for this study. A statement of HAC was noted in the referral for autopsy in 55 subjects. Eighteen subjects had displayed symptoms of PD or PDD/DLB during their lifetime. In addition, there were 27 age matched “control” subjects without registered alcohol consumption or signs of PD or PDD/DLB. In total, there were 72 male and 28 female subjects, with a mean age and standard error of means (SE) at death of 73 ± 1 years. The demographics of the included cases are summarized in Table 1. The use of archived tissue for this study was approved by the local ethical committee (Dnr 2011/286 and updated 2015).

**Table 1:** Demographics

	ALL n (%)	With HAC *n* (%)	Controls *n* (%)	With PD, PDD *n* (%)	Statistics KTW
All	100	55	27	18	–
Post mortem delay in hours, m ± SE	88 ± 5	100 ± 8	76 ± 10	70 ± 7	ns
Male/female	72/28	42/13	19/8	11/7	–
age at death, m ± SE	73 ± 1	72 ± 1	72 ± 1	74 ± 2	ns
Brain weight in grams, m ± SE	1393 ± 14	1378 ± 17	1449 ± 26	1350 ± 35	ns
With dementia	20 (20)	5 (9)	0 (0)	15 (88)	–

*n* number, *HAC* heavy alcohol consumption, *PD* Parkinson Disease, *PDD* PD with dementia (HAC not registered), *m ± SE* mean ± standard error of means, *KWT* Kruskal–Wallis Test

At autopsy, the brains were weighed and fixated in 10% buffered formalin. After fixation, they were cut into one-centimeter-thick coronal slices and assessed for macroscopic lesions. Samples for microscopic examination were taken according to a standardized protocol from 16 regions (Supplement Table 1). These tissue samples were placed in mega cassettes and placed in fixative. The total fixation time was approximately 2 weeks. From each block, sections, seven μm in thickness, were cut and stained with hematoxylin–eosin (HE). The antibodies used for IHC stains were applied in automatic stainers on defined sections (Supplement Table 1). Details regarding the methods used for the IHC stains for visualization of the altered proteins have been published previously [2, 4, 5]. A liver sample was obtained at autopsy and placed in a cassette and fixed in formalin. Four μm thick sections of the liver sample were cut and stained with HE, van Gieson (VG) stains and IHC applying antibodies directed to αS (Supplement Table 1).

**Fig. 1:**
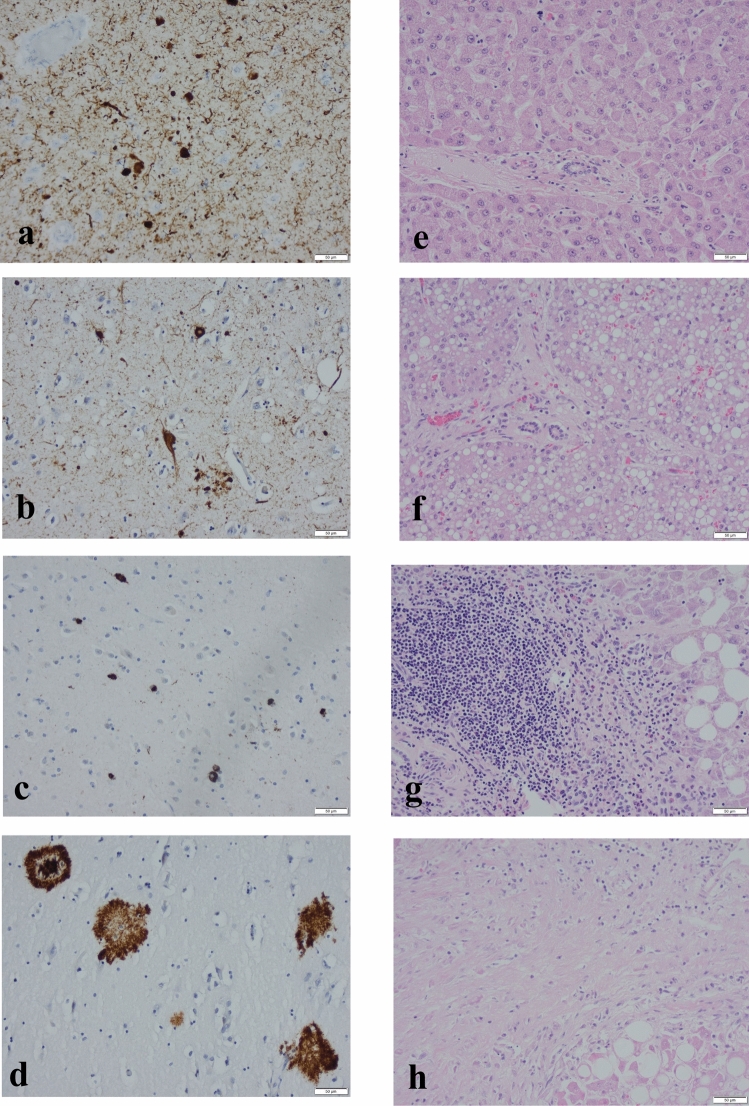
Photomicrographs of stained section of brain region amygdala (**a**–**d**) and liver (**e**–**h**). **a** immunohistochemically stained with antibodies (ab) directed to α-synuclein the labeling is seen in brown, **b** ab directed to hyperphosphorylated τ, **c** ab directed to amyloid β-protein, **d** ab directed to transactive DNA binding protein 43 **e** hematoxylin-eosin stained normal liver **f** steatosis and some fibrosis **g** inflammation and steatosis **h** cirrhosis. Magnification × 200 (bar 50 μm)

The protein alterations to be seen in the brain (Fig. 1a–d) were assessed following the recommended consensus criteria summarized in Table 2. Liver sections were evaluated in HE, VG, and IHC/αS stains using light microscopy at magnifications ranging from × 20 to × 100. The LP was assessed following published criteria (Fig. 1e–h), modified for postmortem tissue, as seen in Table 3. A total score for LP was calculated, i.e., a sum of the scores for steatosis (0–3), inflammation (0–4), and fibrosis (0–4), ranging from 0 to 11. A score of ≤ 2 was assigned as none to mild LP, 3–5 as moderate LP, and ≥ 6 as severe LP. The presence or absence of IHC/αS in liver tissue was also assessed.

**Table 2:** Altered proteins visualized using immunohistochemistry; the extent assessed based on the regional distribution as recommended

Altered protein	Criteria	Stage	Altered protein seen in the section of
hyperphosphorylated τ	Braak stage [34]	I	Hippocampus, transentorhinal region
		II	Hippocampus, entorhinal region
		III	Hippocampus, occipito-temporal gyrus
		IV	Temporal cortex, medial gyrus
		V	Occipital cortex, peristriatal area 19
		VI	Occipital cortex, para- and striatal areas 17.18
amyloid β protein	Thal phase [35]	1	Neocortex
		2	Hippocampus
		3	Amygdala, striatum
		4	Midbrain
		5	Cerebellum
α synuclein	Braak stage [33]	1	Medulla, dorsal nucleus of vagus
		2	Pons, locus coeruleus
		3	Midbrain, substantia nigra
		4	Amygdala
		5	Cingulate gyrus, temporal cortex
		6	Frontal, parietal cortex
transactive DNA binding protein 43	Josephs phase [28]	1	Amygdala
		2	Hippocampus, entorhinal
		3	Hippocampus, dentate layer
		4	Occipito-temporal, inferior temporal
		5	Frontal cortex

**Table 3:** Liver pathology

Alteration	Criteria	Stage	Extent of alterations
steatosis	Goodman [38]	0	< 5%
		1	5–33%
		2	33–66%
		3	66%
inflammation	Batts and Ludvig [36]	1	Portal, minimal
		2	+ periportal light
		3	+ periportal moderate
		4	+ septal
fibrosis	Batts and Ludvig [36]	0	None
		1	Portal
		2	Periportal
		3	Septal
		4	Cirrhos

Statistical analyses were performed using SPSS, applying non-parametric tests. For descriptive statistics, the mean ± standard error of means (m ± SE) was calculated. Statistical differences between the studied groups were assessed using the Mann Whitney *U* (MWU) and Kruskal–Wallis test. For categorical data, Fischer’s exact test and Pearson Chi-Squire (PCS) test were used, and for correlation between the studied variables, the nonparametric Spearman correlation test was used.

## Results

### Liver pathology

**Fig. 2:**
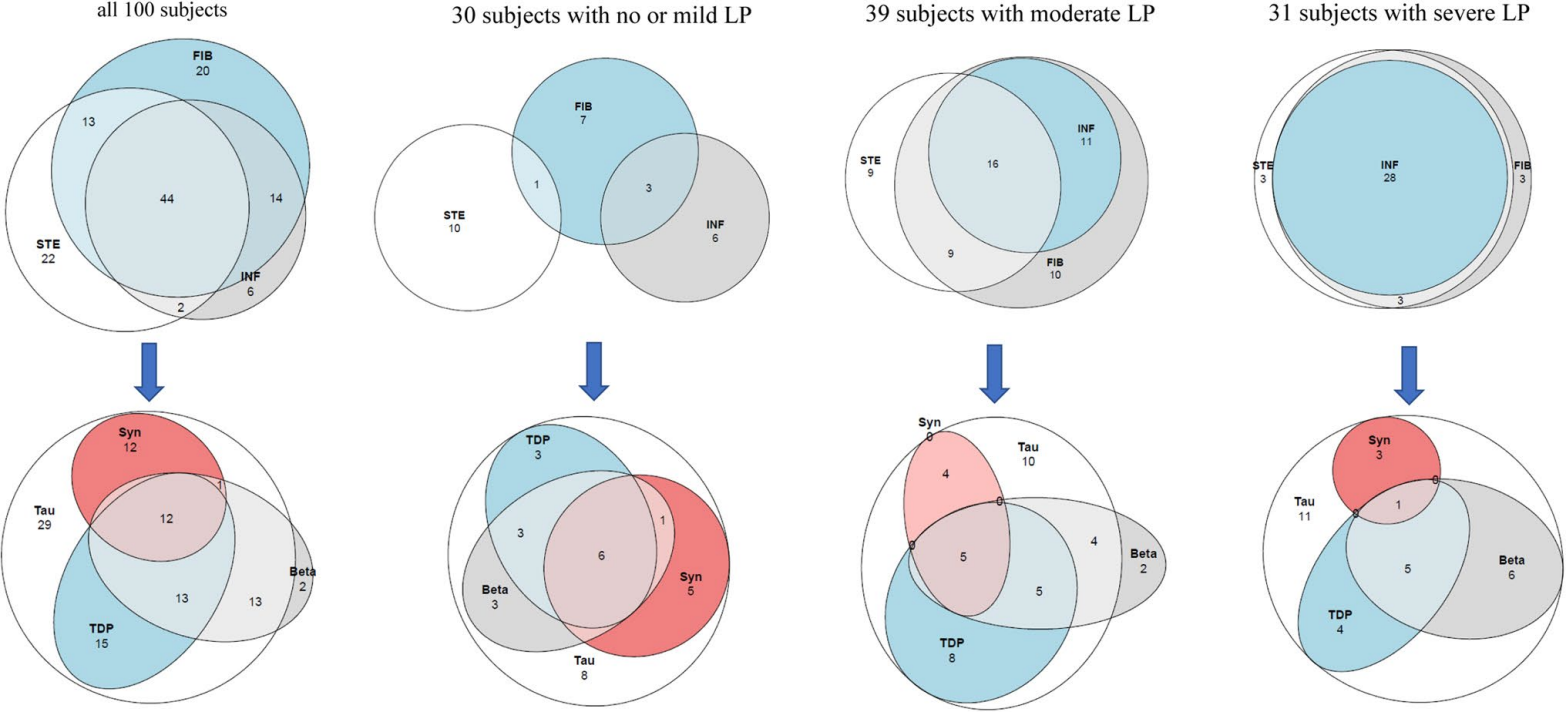
Venn diagram. Upper row *LP* liver pathologies, *STE* steatosis not seen in 32% of subjects, *INF* inflammation, not seen in 34% of subjects, *FIB* fibrosis, not seen in 22% of subjects. Lower row, brain pathologies, *Tau* hyperphoshorytaled τ, not seen in 5% of subjects, *Beta* amyloid β -protein, not seen in 59% of subjects, *TDP* transactive DNA binding protein 43, not seen in 60% of subjects, *SYN* α-synuclein, not seen in 73% of subjects. Five subjects lacked any LP and 5 subjects lacked any brain pathology (not the same subjects)

The extent of LP is summarized for all 100 subjects in Table 4. There were five subjects lacking any liver alterations. These subjects are included in the group of those with mild LP in Tables 4 and 5. There were 32 subjects that lacked any signs of steatosis, 34 lacked signs of inflammation, and 22 lacked signs of fibrosis in their liver tissue. In 25 subjects, mild (score from 1–2) LP was noted, while 39 had moderate (score 3–5), and 31 had severe (score 6–10). Steatosis, inflammation, and fibrosis were most often seen in subjects with registered HAC, and the extent of LP was most severe and significantly higher (Mann–Whitney *U* test, *p* < 0.001) in the HAC group (mean ± SE 4.7 ± 0.3) compared to subjects with PD, PDD/DLB (mean ± SE 2.6 ± 0.5). In Fig. 2, a Venn diagram visualizes the incidence of concomitant LPs.

**Table 4:** The incidence and extent of liver pathology

	ALL *n* (%)	With no, or mild, liver pathology	With moderate liver pathology	With severe liver pathology	Statistics PCS
All	100	30	39	31	
Age at death, m ± SE	73 ± 1	72 ± 2	74 ± 1	72 ± 1	ns
Male/female	72/28	19/11	30/9	23 / 8	ns
With dementia	20 (20)	9 (30)	7 (20)	4 (13)	ns
With liver steatosis^1^	68 (68)	10 (33)	27 (69)	31 (100)	< 0.001
Mild steatosis, 1^2^	25 (37)	7 (70)	14 (52)	4 ( 13)	
Moderate steatosis, 2^2^	22 (32)	3 (30)	9 (33)	10 ( 32)	< 0.001
Severe steatosis, 3^2^	21 (31)		4 (15)	17 ( 55)	
With liver inflammation^1^	66 (66)	9 (30)	29 (74)	28 ( 90)	< 0.001
Portal inflammation, 1^2^	30 (45)	8 (89)	13 (45)	9 ( 32)	
Light periportal inflammation, 2^2^	26 (40)	1 (11)	12 (41)	13 ( 46)	< 0.001
Moderate periportal inflammation, 3^2^	10 (15)		4 (14)	6 ( 22)	
With liver fibrosis^1^	78 (78)	10 (33)	37 (95)	31 (100)	< 0.001
Portal fibrosis, 1^2^	15 (19)	7 (70)	8 (22)		
Periportal fibrosis, 2^2^	41 (53)	3 (30)	26 (70)	12 (38)	
Septal fibrosis, 3^2^	14 (18)		3 (8)	11 (36)	< 0.001
Cirrhosis, 4^2^	8 (10)			8 (26)	
Score of liver pathology, m ± SE	4.2 ± 0.3	1.2 ± 0.1	4.2 ± 0.1	7.0 ± 2.2	KWT 0.000
Range of liver pathology	0–10	0–2	3–5	6–10	

*n* number, *m ± SE* mean ± standard error, *KWT* Kruskal–Wallis Test

^1^percentage of alteration counted in relation to the total number of subjects

^2^percentage of the extent of alteration in relation to the number of subjects with the alteration. Statistics PCS, Pearson’s Chi-Squire

**Table 5:** Incidence and extent of altered proteins and astrocytic pathology in the brain

	ALL *n* (%)	With no, or mild, liver pathology	With moderate liver pathology	With severe liver pathology	Statistics
All	100	30	39	31	
with HPτ	95 (95)	29 (97)	36 (92)	30 (100)	ns
HPτ with a distribution as seen in PSP or AgD	2 (2)		1 (3)	1 (3)	
Only seen in locus coeruleus	20 (20)	3 (10)	12 (33)	5 (17)	ns
HPτ, Braak stage I-II	50 (50)	18 (60)	16 (44)	16 (53)	
HPτ, Braak stage III-IV	22 (22)	8 (27)	7 (20)	7 (23)	
HPτ, Braak stage V-VI	1 (1)			1 (3)	
With Aβ	41 (41)	13 (43)	16 (41)	12 (39)	ns
With Aβ, Thal phase 1–2	17 (41)	4 (31)	7 (44)	6 (50)	ns
With Aβ, Thal phase 3–4	24 (59)	9 (69)	9 (56)	6 (50)	
With TDP43	40 (40)	12 (40)	18 (46)	10 (32)	ns
TDP43 with a distribution as seen in FTLD	1 (3)	1 (8)			
With TDP43, Josephs stage 1–2	35 (87)	9 (75)	17 (94)	9 (90)	ns
With TDP43, Josephs stage 3–4	4 (10)	2 (17)	1 (6)	1 (10)	
With αs	27 (27)	12 (40)	10 (26)	5 (16)	ns
With αs, stage 1–2	4 (15)	2 (17)	2 (20)		
With αs, stage 3	1 (4)		1 (10)		
With αs, stage 4	3 (11)				< 0.050
With αs, stage 5–6	19 (70)	10 (83)	7 (70)	5 (100)	
With Alzheimer type II astrocytes	27 (27)	2 (7)	8 (21)	17 (55)	< 0.001
ARTAG	29 (29)	7 (23)	10 (26)	12 (39)	ns

*n* number, *m ± SE* mean ± standard error, *HPτ* hyperphosphorylated τ, *Aβ* amyloid β-protein, *TDP43* transactive DNA binding protein 43, *αS* α synuclein, *PSP* Progressive Supranuclear Palsy, *AgD* Argyrophilic Grain Disease, *FTLD* Frontotemporal Lobar Degeneration, *ARTAG* Age Related Tau AstroGliopathy, *PCS* Pearson’s Chi-Squire

### Brain pathology

Table 5 summarizes the incidence and extent of altered proteins in the 100 subjects, with 30 having mild, 39 moderate, and 31 severe LP. Five subjects lacked HPτ pathology in their brains, 59 subjects lacked Aβ, 60 lacked TDP43, and 73 lacked αS pathology in their brains. Overall, most subjects displayed HPτ pathology at Braak stage I–II, while only one subject, who had severe LP, displayed a high level of HPτ pathology. Forty-one percent of the subjects displayed Aβ pathology, with 59% of them in Thal phase 3–4. TDP43 pathology was observed in 40% of the subjects, with 87% of them displaying TDP43 pathology in Josephs stage 1–2. αS pathology was observed in 27% of the cohort, with a substantial number, 70%, of these subjects displaying severe αS pathology (selection bias). In Fig. 2, a Venn diagram visualizes the incidence of concomitant pathologies. All altered proteins were concomitantly observed in 12% of all subjects, in 20% of those with mild LP, 13% with moderate LP, and in 3% of those with severe LP. Vascular tissue alterations were seen in 36% of the whole cohort, with more severe lesions in 19% of subjects (both macroscopic and microscopic lesions). Cerebral amyloid angiopathy was noted in 20% of subjects. There was no correlation between these lesions and LP.

### Glial pathology in the brain

In 27 subjects out of the whole cohort, Alzheimer’s type II astrocytes were seen (Table 5). They were present in 55% of subjects with severe LP, compared to 7% of the 25 subjects with mild LP, and were never observed in the five subjects without any liver alterations. The mean ± SE of LP was significantly higher (Mann Whitney *U* test, *p* < 0.001) in subjects with Alzheimer’s type II astrocytes (6.1 ± 0.5) compared to subjects without Alzheimer’s type II astrocytes (3.4 ± 0.2). There was a significant negative association (Fisher’s exact Test 0.04) between the observation of Alzheimer’s type II astrocytes and αS pathology in the brain. Age-related Tau Astrogliopathy (ARTAG) was seen in 29% of all subjects. ARTAG was found in 39% of subjects with severe LP, 26% with moderate, 24% in subjects with mild LP, and 20% of subjects without alterations related to LP. The extent of LP did not differ significantly between subjects with (4.7 ± 0.4) or without (3.9 ± 0.3) ARTAG. ARTAG was significantly associated with Alzheimer’s type II astrocytes (Fisher’s Exact Test, *p* = 0.01).

### Dementia

Twenty subjects, comprising 12 males and eight females, with a mean age ± SE at death of 73 ± 2 years, had displayed cognitive impairment during their lifetime. Among these 20 subjects with dementia, nine displayed none to mild LP (30% of all in this group), seven displayed moderate LP (23% of all in this group), and four displayed severe LP (13% of all in this group). Out of the 20 demented subjects, all displayed mixed pathology. In 15 subjects, cognitive decline was attributed to αS pathology. Among these 15 subjects with αS pathology, concomitant alterations included Primary Age-Related Tauopathy (PART) in seven subjects, concomitant ADNC in one subject, PART and LATE in one subject, and ADNC and LATE in six subjects. In four subjects, cognitive decline was attributed to ADNC with concomitant LATE, and in one subject to FTLD-TDP with PART.

### Relationship between brain and liver pathology

When looking at the contingency between the brain and liver alterations in the whole group of 100 subjects, it was observed that there was a significant negative correlation between liver steatosis and both αS and Aβ in the brain (Table 6). In line with the above, there was a significant negative correlation between liver fibrosis and αS in the brain. Conversely, there was a significant positive correlation between liver inflammation and Aβ in the brain. However, when the study group was divided based on the severity of LP, no significant associations were found.

**Table 6:** Nonparametric Spearman’s rho (r) correlation of categorical data, for all 100 subjects included

Liver alteration	with Aβ	with αS	Alz II
With steatosis	− 0.21^1^	− 0.31^2^	0.27^2^
With inflammation	0.21^1^		0.20^1^
With fibrosis		− 0.28^2^	0.27^2^

Only significant associations listed ^2^0.01, ^1^0.05

*HPτ* hyperphosphorylated τ, *Aβ* amyloid β-protein, *TDP43* transactive DNA binding protein 43, *αS* α synuclein, *Alz II* Alzheimer type II astrocytes in the brain

When assessing correlations between the extent of brain and LP (Table 7) across all 100 subjects, a significant correlation was observed between different altered proteins seen in the brain and various liver alterations. Specifically, a significant negative correlation was found between the extent of steatosis and the extent of αS pathology, between the extent of fibrosis and the extent of αS pathology, and between the extent of LP and the extent of αS pathology. Most correlations prevailed when the assessment was carried out on males/females or subjects with and without dementia (Table 7). Additionally, a significant negative correlation was observed between the extent of liver fibrosis and TDP43, but only in males, and a significant positive correlation between liver inflammation and TDP43 in the brain, but only in individuals with dementia.

**Table 7:** Nonparametric Spearman’s rho (r) correlations between the extent of assessed pathologies

	All	Male	Female	Without dementia	With dementia
Number	100	72	28	80	20
HPτ and Aβ	0.47^2^	0.45^2^	0.54^2^	0.45^2^	
HPτ and αS	0.35^2^	0.34^2^	0.38^1^	0.32^2^	
HPτ and TDP43	0.45^2^	0.49^2^		0.38^2^	0.48^2^
Aβ and TDP43	0.45^2^	0.52^2^		0.34^2^	0.56^2^
Aβ and αS		0.26^1^			
αS and TDP43				0.24^1^	
Steatosis and fibrosis	0.47^2^	0.49^2^	0.38^1^	0.42^2^	0.53^1^
Steatosis and LP	0.75^2^	0.74^2^	0.74^2^	0.72^2^	0.75^2^
Inflammation and fibrosis	0.32^2^	0.30^1^		0.31^2^	
Inflammation and LP	0.61^2^	0.52^2^	0.75^2^	0.61^2^	0.68^2^
Fibrosis and LP	0.82^2^	0.86^2^	0.76^2^	0.82^2^	0.86^2^
αS and fibrosis	− 0.29^2^	− 0.28^1^	− 0.44^1^		
αS and steatosis	− 0.29^2^				
αS and LP	− 0.28^2^	− 0.24^1^	-0.39^1^		
TDP43 and inflammation					0.45^1^
TDP43 and fibrosis		− 0.27^1^			

Correlation shown when significance < 0.05^1^, < 0.01^2^

*HP τ* hyperphosphorylated τ, *Aβ* amyloid β-protein, *αS* α-synuclein, *TDP43* transactive DNA binding protein 43, *LP* liver pathology (see Table 5)

## Discussion

In our analysis of 100 subjects, we observed a negative correlation between αS and steatosis, but also a significant negative correlation between liver fibrosis and αS in the brain. When assessing correlations between the extent of pathologies, a significant negative correlation was noted between the extent of LP, liver steatosis, or fibrosis, and the extent of αS in the brain. Thus, both categorical data and the extent of pathologies revealed a negative correlation between LP and αS pathology. Furthermore, there was a significant negative association between the observation of Alzheimer type II astrocytes and αS pathology in the brain. Based on our results, i.e., a negative correlation between LP and αS in the brain, it seems that subjects with LP are not predisposed to develop PD, PD /DLB; rather, the opposite. This outcome is in line with the recent large meta-analysis reporting an inverse association between alcohol consumption and PD [22, 37]. The etiopathogenesis of this outcome is unclear. Gastrointestinal tract is altered in subjects with HAC and it has been reported that moderate acute alcohol consumption immediately damages the enterocytes [12, 15, 45]. In parallel αS is observed in the gastrointestinal nervous system years prior to be seen in the brain [25]. Thus, the question does arise whether eventual alcohol-related alterations in the gastrointestinal tract influence the development of αS related alterations seen in the neuronal cell population of the gut. Moreover, it is well known as was also seen by us that in subjects with HAC the glial cells are altered in the brain but nothing is known regarding the glial cell population in the gut. Furthermore, it is not clear how severe LP might change livers role in the suggested clearance of αS [47].

In our analysis, we found no association between HPτ and any of the assessed liver pathologies. However, there was a significant negative correlation between liver steatosis and Aβ. Here, we did not identify any association between various extents of LP and the extent of Aβ in the brain. The latter finding is certainly in line with prior research that has reported no significant influence of HAC on Aβ [1]. It has been suggested that beer drinkers may have lower levels of Aβ in their brains, but the assessment of the extent of Aβ in the referred study was not based on the regional distribution of Aβ as in the current study but on the extent seen in one cortical section [28]. In contrast, recent animal studies have suggested that nonalcoholic liver steatosis promotes Aβ accumulation in the brain [44]. Consistent with the findings reported by Peng and colleagues, a clinical study assessing nonalcoholic fatty liver disease and plasma and imaging biomarkers of AD and vascular brain lesions suggested a link between midlife nonalcoholic fatty liver disease and dementia [33]. Noteworthy, the results obtained by us, Peng and colleagues in 2024 and Lu and colleagues in 2024 are not as such comparable as the methods differ significantly. We did see Aβ more frequently in the brains of subjects with liver inflammation compared to subjects without this liver alteration; however, the number of subjects with liver inflammation in our study was limited. Moreover, none of the assessed liver alterations influenced the severity of Aβ in the brain. This observation is in line with experimental studies suggesting that LP primarily influences glial cells (microglia) rather than Aβ accumulation [17]. Thus, based on our results, we cannot confirm the hypothesis that LP, i.e., steatosis, inflammation, or fibrosis, indeed influences the development of ADNC, i.e., HPτ and Aβ.

In 2020, in a report of the Lancet Commission discussion dementia prevention, intervention and care have listed alcohol consumption as a risk for dementia [32]. Noteworthy, references listed in this publication are various clinical studies as reports including neuropathological observations are scarce. In 2009, Clive Harper and in 2014 Suzanne de la Monte and Gillian Krill described and summarized alcohol-related brain damage and in both these publications the main alteration given is cell loss in various brain locations [16, 19]. We did not assess cell loss that eventually can be seen whereas we centered on protein alterations that are not described previously by others.

We created a Venn diagram to visualize the incidence of altered proteins in the brain among subjects with varying degrees of LP severity. Noteworthy, the number of subjects with concomitant alterations decreased significantly from 20% in those with mild LP to 3% in subjects with severe LP. Age at death is a significant factor in the observation of concomitant pathologies and it did not differ significantly between subjects with mild, moderate or severe LP. Thus, the difference cannot be attributed solely to the age of the subjects [3]. Noteworthy, dementia was registered in 30% of subjects with mild LP when compared with 13% of subjects with severe LP. Our observation, confirms that further studies on the brains of individuals with a history of HAC are warranted. Does severe LP indeed alter significantly some of the altered proteins associated with cognitive impairment?

Assessing alterations in peripheral tissues parallel to the evaluation of pathologies in the brain is of great interest. Previously, assessments of cardiovascular pathologies, as well as those of the kidney and pancreas, have been conducted to verify or deny existing associations between protein alterations in the brain and systemic diseases. Noteworthy, many of the proposed associations based on clinical or animal studies have not been confirmed, i.e., cardiovascular disease and diabetes do not appear to influence the extent of altered proteins in the brain, whereas these systemic diseases can certainly lead to vascular tissue damage in the brain [4, 21, 30].

The liver tissue was significantly affected in subjects with HAC; in individuals with severe LP, Alzheimer’s type II astrocytes were seen more frequently compared to controls. Astrocytes have been implicated in being of significance for Aβ processing [49, 53, 54]. In our 31 subjects with severe LP and frequently encountered Alzheimer type II astrocytes, however, no significant association was observed between the extent of LP and brain pathologies. The recently defined astrocytic alteration, ARTAG, i.e., glial HPτ pathology, was not influenced by LP. A significant association was, however, noted between Alzheimer’s type II astrocytes and ARTAG.

Studies integrating assessment of the brain and various peripheral organs are generally difficult to carry out. An autopsy is seldom performed [50]. Moreover, in many centers, especially when dealing with brain alterations, a brain-only autopsy is the preferred approach, i.e., the Netherlands Brain Bank. A long postmortem delay, often up to 240 h, is not unusual and can lead to tissue alterations that influence the assessment options. Thus, some cases may need to be excluded, particularly when peripheral organs such as the pancreas, gut and liver are included. Finally, and maybe most importantly, the visualizing techniques vary substantially. Some laboratories implement IHC, while others use in situ hybridization. Moreover, various antibodies and techniques (manual or various automated) are used. Thus, comparing results obtained by different laboratories is indeed difficult, if not impossible. Here, we assessed the brain pathology and the LP in a standardized manner (Tables 2 and 3) and noted that, as expected, the extent of all altered proteins in the brain correlated significantly and strongly with each other. Similarly, a significant correlation was noted between the different liver alterations. What has been demonstrated here emphasizes that human studies are both possible and informative, but they also present many pitfalls and challenges.

The overall sample size, whether it is 1000, 100, or 10 subjects, is certainly of significance. It has previously been reported that selection bias might alter the outcome [42]. Thus, here, we prefer to consider the results obtained from all 100 subjects to be reliable, while the outcomes when the cohort is separated based on gender or dementia are considered less reliable. When analyzing the data obtained from all 100 subjects with various extents of liver damage (LP ranging from 0 to 10), we noticed a negative correlation regarding the categorical data between liver steatosis and αS, we detected a significant negative correlation between the extent of liver steatosis and fibrosis and the extent of αS in the brain, we noticed a significant negative association between the observation of Alzheimer type II astrocytes and αS pathology in the brain, we detected a negative correlation regarding the categorical data between liver steatosis and Aβ and we noted no significant associations between LP and HPτ or TDP43.

## Conclusion

Here we observed a negative correlation both regarding categorical data and the extent of alteration, between αS and LP, a significant negative association between the observation of Alzheimer type II astrocytes and αS pathology in the brain. These observations are in line with the recently published large meta-analysis suggesting an inverse association between alcohol consumption and PD. Further, we observed a negative correlation of categorical data between steatosis and Aβ. Contrary, the most common aging-related alteration, HPτ and TDP43 were not influenced by LP. In 20% of subjects with no or mild LP mixed pathologies were seen (HPτ + Aβ + TDP43 + αS) when compared with in 3% of those with severe LP. In line with the above 30% of subjects with no or mild LP were demented when compared with 13% of those with severe LP. One needs to be aware of all pitfalls and challenges associated with the analysis of postmortem human tissue and one must consider the various methods and assessment strategies used.
